# Prophylactic effect of pilocarpine on acute sialadenitis following radioactive iodine therapy in thyroid cancer patients

**DOI:** 10.7150/ijms.84590

**Published:** 2024-01-01

**Authors:** Eun Kyoung Choi, Jin Kyoung Oh, Yong-An Chung, Hyeonseok Jeong, Hoon Choi, Kwanhoon Jo

**Affiliations:** 1 Department of Radiology, Incheon St. Mary's Hospital, College of Medicine, The Catholic University of Korea, Seoul, Republic of Korea.; 2 Department of Surgery, Incheon St. Mary's Hospital, College of Medicine, The Catholic University of Korea, Seoul, Republic of Korea.; 3 Division of Endocrinology & Metabolism, Department of Internal Medicine, Incheon St. Mary's Hospital, College of Medicine, The Catholic University of Korea, Seoul, Republic of Korea.

**Keywords:** Thyroid Neoplasms, Pilocarpine, Sialadenitis, Pre-Exposure Prophylaxis, Radioactive Iodine

## Abstract

**Purpose:** Our aim was to evaluate the effect of prophylactic pilocarpine on acute salivary symptoms after radioactive iodine (RAI) therapy in patients with differentiated thyroid cancer.

**Methods:** We enrolled 88 patients (76 women and 12 men; mean age: 47 years; range: 20-74 years) with differentiated thyroid cancer who received RAI. Patients were divided into pilocarpine (51 patients) and control (37 patients) groups. Pilocarpine was given orally, at a dose of 5 mg three times a day, from 2 days before and 12 days after RAI therapy. Symptoms and signs of acute sialadenitis within 3 months of RAI therapy were recorded.

**Results:** During the 3 months after RAI therapy, 13 of the 88 patients (14.7%) developed acute symptomatic sialadenitis (swelling or pain of salivary glands). Acute salivary symptoms were reported by 4 (7.8%) and 9 (24.3%) patients in the pilocarpine and control groups, respectively. Acute salivary symptoms were less frequent in the pilocarpine than control group (*p* = 0.04), but did not differ by age, sex, or RAI dose (*p* = 0.3357, *p* = 0.428, and *p* = 0.2792).

**Conclusions:** Pilocarpine reduced the likelihood of acute sialadenitis after RAI therapy in patients with differentiated thyroid cancer.

## Introduction

In patients with differentiated thyroid cancer, radioactive iodine (RAI) is used after total thyroidectomy for adjuvant ablation, treatment of metastases or suspected residual disease, and early detection of disease recurrence based on serum thyroglobulin and/or I-131 scan during follow up 1. RAI is associated with dose-related complications although it is considered to be reasonably safe. Salivary gland damage is the most frequent complication of high-dose RAI, and presents as dry mouth, pain and swelling of the salivary glands, and change of taste 2-4. Oral hydration and chewing candy or gum are frequently advised as methods to prevent salivary gland damage, but there is no evidence of their effectiveness 4.

Pilocarpine is a non-selective muscarinic agonist with β-adrenergic activity that increases salivary secretion and improves xerostomia in patients with functional salivary glands 5. It is used for treating xerostomia after radiotherapy for head and neck cancer, and for symptomatic relief of Sjogren's syndrome 6,7. However, there are only a few studies on the efficacy of pilocarpine for the prophylaxis or treatment of sialadenitis in thyroid cancer patients receiving RAI therapy 8-10. To the best of our knowledge, no previous study has assessed the efficacy of prophylactic pilocarpine alone for preventing acute sialadenitis and salvaging salivary glands after RAI therapy. We evaluated the prophylactic effect of pilocarpine on acute salivary symptoms after RAI therapy in patients with differentiated thyroid cancer.

## Materials and Methods

### Study design and patient population

**Figure 1:**
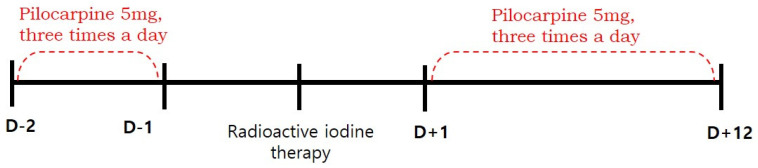
Timeline of treatment received by pilocarpine group.

This retrospective study was approved by the institutional review board of our medical center and the requirement to obtain informed consent was waived. We reviewed the medical records of 102 patients with differentiated thyroid cancer who received RAI therapy after total thyroidectomy between January and June 2016. We excluded patients with pre-existing salivary symptoms or diseases involving the salivary glands (such as rheumatoid arthritis, Sjogren's syndrome, lymphoma, or AIDS), taking xerostomic drugs including anticholinergic, antihistamine, or cardovascular drugs (reserpine, methyldopa, chlorthiazide, furosemide, metoprolol, and calcium channel blockers etc.), and those who had undergone previous radiotherapy of the head and neck. Eighty-eight patients were finally enrolled. The patients were divided into pilocarpine and control groups, according to whether they were contraindicated for pilocarpine. Contraindications included closed-angle glaucoma, iritis, asthma, bronchitis, chronic obstructive pulmonary disease, hepatic disease, and cardiac disease. Fifty-one eligible patients were assigned to the pilocarpine group, and thirty-seven patients with any of the above contraindications were assigned to the control group. Patients in the pilocarpine group received 5 mg of pilocarpine, orally three times a day for 14 days (from 2 days before and 12 days after RAI), whereas patients in the control group received RAI therapy without the administration of any medication 10. (Figure 1). Because of the high iodine intake in South Korea 11, all patients were maintained on a low-iodine diet for at least 2 weeks prior to RAI therapy. Patients in both groups were prepared for RAI with either recombinant human thyroid stimulating hormone (Thyrogen; Sanofi Genzyme, Cambridge, MA, USA) or prolonged thyroid hormone withdrawal, followed by high-dose RAI therapy (≥ 3.7 GBq). After RAI therapy, the patients fasted for 4 hours to ensure complete iodine absorption and reduce the risk of nausea and vomiting. Patients in both groups were advised to use sialogogues (drinking fluids and chewing candy or gum).

### Clinical assessment

Self-reported salivary gland symptoms were recorded using a detailed questionnaire administered in person during a clinical visit 3 month after RAI therapy, or contactless over the phone. The questionnaire consisted of questions regarding swollen, painful salivary gland symptoms of acute obstructive sialadenitis within 3 months of RAI therapy. In addition, those patients with acute sialadenitis were surveyed for the presence of subjective xerostomia. If present, they were followed up for 6 months or longer to see how long the symptoms lasted and to explore the long-term effect of pilocarpine on salivary gland function.

### Statistical analysis

Statistical analyses were performed using Stata software (version 13.1; StataCorp, College Station, TX, USA). Categorical and continuous variables are presented as frequencies with percentages and median with range, respectively. *P*-values < 0.05 were considered statistically significant. Categorical variables were analyzed using chi-square test or Fisher's exact test, and continuous variables using Student's t-test or the Mann-Whitney U test.

## Results

There were no significant differences between the groups in terms of age, sex, preparation method (recombinant human thyroid stimulating hormone vs. thyroid hormone withdrawal), or RAI dose (Table 1). In the overall patients, there was no association between the age and the occurrence of acute symptomatic sialadenitis (*p* = 0.307). Thirteen of the 88 patients (14.7%) had acute symptomatic sialadenitis (swelling or pain of salivary glands) within 3 months of RAI therapy. In the pilocarpine and control groups, acute symptomatic sialadenitis was reported by 4 (7.8%) and 9 (24.3%) patients, respectively (Table 2). Acute symptomatic sialadenitis developed less frequently in the pilocarpine than control group (*p* = 0.04).

Five of the 13 pilocarpine patients with acute symptomatic sialadenitis reported subjective xerostomia, and one patient reported loss of taste (Table 3). Four patients in the control group, and one patient in the pilocarpine group complained of xerostomia with swollen or painful salivary gland. Four of these patients reported persistent xerostomia more than 6 months later. One patient in the control group complained of loss of taste, and her symptom was resolved within 6 months of RAI therapy.

Adverse effects of pilocarpine, such as sweating, rhinitis, nausea, urinary frequency, dizziness, chills, and asthenia, were not experienced by any patients.

## Discussion

In this retrospective study of thyroid cancer patients who received high-dose RAI, 14.7% of patients developed radiation-induced acute sialadenitis and the prophylactic use of pilocarpine improved patient symptoms. Pilocarpine proved to be an efficacious sialagogue for preventing acute radiation-induced sialadenitis.

Because of the cholinergic effects of pilocarpine, it has been used to relieve the symptoms of acute and chronic sialadenitis (xerostomia and pain or swelling of the salivary glands) caused by RAI therapy 8,9. In this study, prophylactic administration of pilocarpine decreased the incidence of acute sialadenitis. Oral pilocarpine might play an important role in preventing salivary gland damage, increasing salivary secretion, and discharging RAI from the salivary glands. However, a previous study did not find any difference in the incidence of acute sialadenitis with versus without the use of oral pilocarpine 10. The lack of efficacy of pilocarpine in the previous study may be explained by the use of high-dose dexamethasone in addition to pilocarpine in patients before and after RAI therapy to prevent vomiting. The use of dexamethasone also explains the lower incidence of sialadenitis reported in the previous study (6.6%) compared with other investigations (11.5-86%) 12,13. Although there are no well-designed trials on the efficacy of dexamethasone for preventing or treating sialadenitis in thyroid cancer patients after RAI therapy, it is likely that the use of high-dose steroids reduces inflammation and the incidence of acute sialadenitis. The previous study evaluated the effect of pilocarpine with dexamethasone, rather than pilocarpine alone.

Pilocarpine is approved by the US Food and Drug Administration for the treatment of radiation-induced xerostomia, and is widely used for treating symptomatic radiation-induced sialadenitis in patients with head and neck cancer 14,15. Despite the efficacy of pilocarpine for the treatment of sialadenitis, studies on the prevention of radiation-induced xerostomia using pilocarpine in patients with head and neck cancer have reported conflicting results 16-19. In patients with head and neck cancer whose salivary glands are irradiated, prophylactic use of pilocarpine improves objective salivary gland function and stimulates saliva production, but does not improve xerostomia symptoms 16. In patients with head and neck cancer, the symptoms of mucositis are common and difficult to distinguish from those of hyposalivation. In our study, none of the patients had mucositis; therefore, it was possible to evaluate the efficacy of prophylactic pilocarpine for preventing acute sialadenitis.

It is important to prevent acute sialadenitis because of the high prevalence of chronic salivary gland dysfunction in patients with a history of the condition 20. Acute sialadenitis is characterized by episodic or permanent inflammation of the salivary glands, especially more frequently in the parotid glands 21. Radiation-induced cellular damage to the salivary glands causes obstructive sialadenitis, presenting as swelling or pain of the affected salivary gland, along with xerostomia or taste alteration. Occasionally, progression of salivary gland dysfunction occurs even several years after RAI. In this study, 30% of acute sialadenitis patients, complained of xerostomia persisting for more than 6 months, and further studies are needed to recommend prophylactic use of pilocarpine for preventing chronic sialadenitis.

There were several limitations to our study. First, it was susceptible to selection bias because of the retrospective design. Second, salivary flow was not assessed objectively. There have been reports of a mismatch between subjective and objective measures of salivary flow 16,20. Therefore, further studies should use subjective and objective methods, such as measurement of saliva flow and imaging, to assess the effects of prophylactic pilocarpine on salivary flow. In addition, the patients included in our study spanned a wide range of age groups. In the analysis aimed at overcoming this limitation, no correlation was observed between age and the incidence rate of acute symptomatic sialadenitis.

In conclusion, prophylactic administration of pilocarpine before and during RAI therapy reduced the incidence of acute radiation-induced sialadenitis in patients with differentiated thyroid cancer.

## Figures and Tables

**Table 1:** Patient characteristics (N = 88).

Characteristics	Pilocarpine group	Control group	*P-*value
Age (years)	44	51	0.3357
Sex, n (%)			0.428
Males	8 (16%)	4 (11%)	
Females	43 (84%)	33 (89%)	
Preparation method, n (%)			0.068
THW	6 (12%)	10 (27%)	
rhTSH	45 (88%)	27 (73%)	
RAI dose (GBq) Mean ± standard deviation	4.5 ± 0.9	4.3 ± 0.9	0.2792

THW, Thyroid hormone withdrawal; rhTSH, Recombinant human thyroid stimulating hormone; RAI, Radioactive iodine

**Table 2:** Acute salivary gland symptoms after radioactive iodine therapy.

Symptoms	Pilocarpine group	Control group	*P*-value
Pain/swelling of salivary glands	4	9	0.04
No symptom	33	42	

**Table 3:** Characteristics of acute sialadenitis patients with xerostomia or taste change (n = 6).

Pilocarpine group	Control group
Female/43, persistent xerostomia	Female/33, persistent xerostomia Female/51, xerostomia, resolved within 6 months Female/54, persistent xerostomia Female/51, persistent xerostomia Female/46, taste change, resolved within 6 months
